# Supplementary material for: Protein Thermal Stability Changes Induced by the Global Methylation Inhibitor 3-Deazaneplanocin A (DZNep)
Source: Biomolecules. 2024 Jul 9;14(7):817. doi: 10.3390/biom14070817 (PMC11274605; doi:10.3390/biom14070817)
Supplement: Supplementary file 1 [file biomolecules-14-00817-s001.zip › Supplementary Materials.pdf]

Supplementary Materials for

Protein Thermal Stability Changes Induced by the Global Methylation Inhibitor 3-Deazaneplanocin A (DZNep)

Christine A. Berryhill [1], Emma H. Doud [1,2,3], Jocelyne N. Hanquier [1], Whitney R. Smith-Kinnaman [1,2], Devon L. McCourry [1,3], Amber L. Mosley [1,2,3,4], Evan M. Cornett [1,3,4,*]

[1] Department of Biochemistry and Molecular Biology, Indiana University School of Medicine, Indianapolis, IN 46202, USA
[2] Center for Proteome Analysis, Indiana University School of Medicine, Indianapolis, IN 46202, USA
[3] Indiana University Simon Comprehensive Cancer Center, Indiana University School of Medicine, Indianapolis, IN 46202, USA
[4] Center for Computational Biology and Bioinformatics, Indiana University School of Medicine, Indianapolis, IN 46202, USA

* Correspondence: evcorn@iu.edu

The PDF file includes:

Supplementary Figure S1: DZNep treatment perturbs the lysine methylome.

Supplementary Figure S2: Transcriptome and proteome replicates show a high level of reproducibility.

Supplementary Figure S3: GO term analysis and correlation between RNAseq and proteome abundances.

Supplementary Figure S4: ATP binders and methyltransferases are more likely to have either altered protein abundance or thermal stability

Supplementary Figure S5: Methylated proteins are affected by DZNep treatment.

Other Supplementary Material includes the following:

Supplementary Table S1: RNAseq data

Supplementary Table S2: Global Proteomics and PISA data

Supplementary Table S3: Summary of Protein and PISA data

Supplementary Table S4: Lysine Methylation Peptide data

Supplementary Table S5: Summary of Lysine Methylation data

Supplementary Table S6: Detected Kme sites

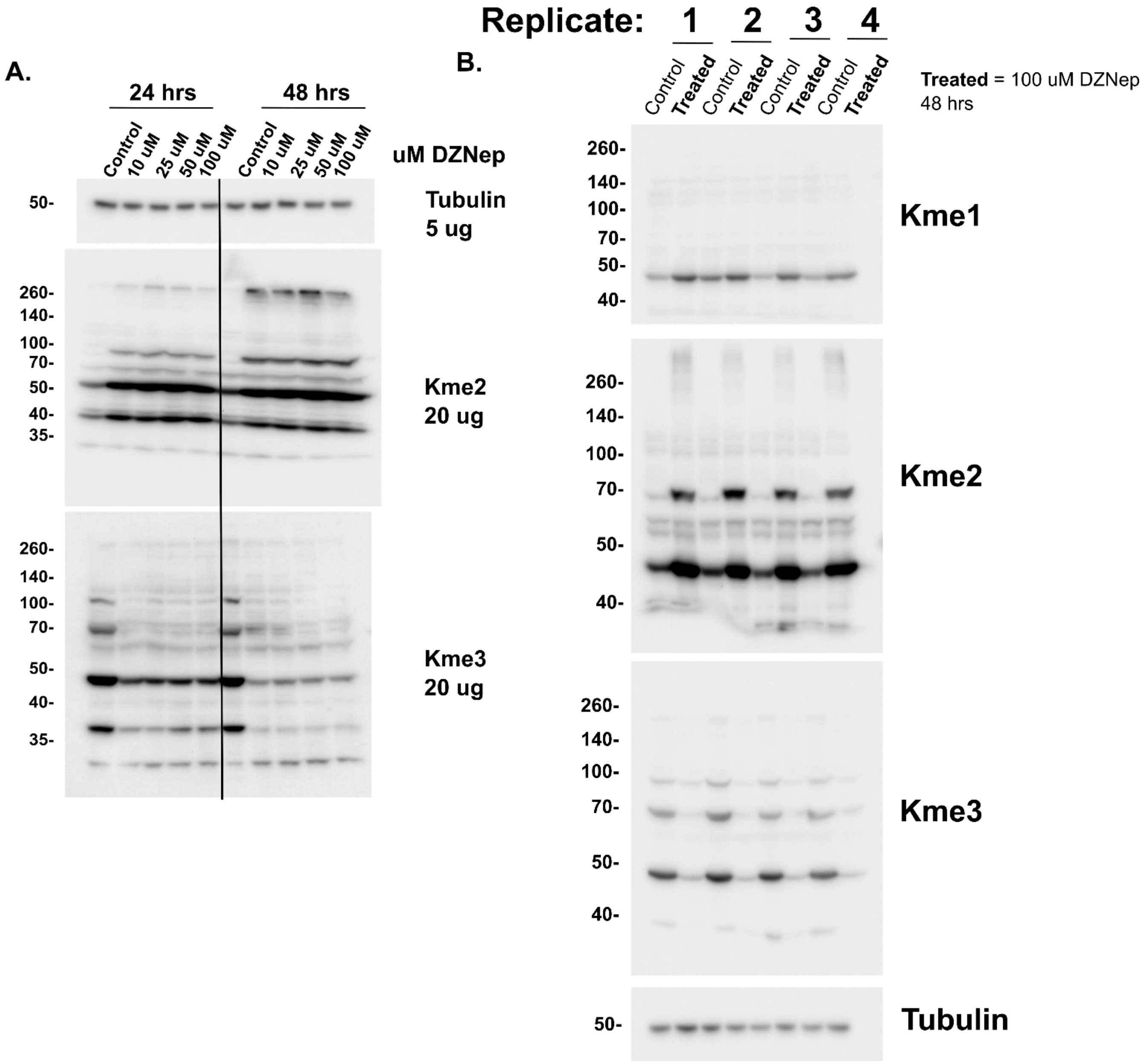

Figure S1: DZNep treatment perturbs the lysine methylome.

(a) Western blot visualizing the impact various concentrations and time has on lysine mono-, di-, and tri-methylation, as indicated. (b) Western blot of the lysine methylome of the lysed global samples. Replicates and drug treatment are indicated across the top. Methyl state is indicated along the side. Blots shown are all run on independent gels.

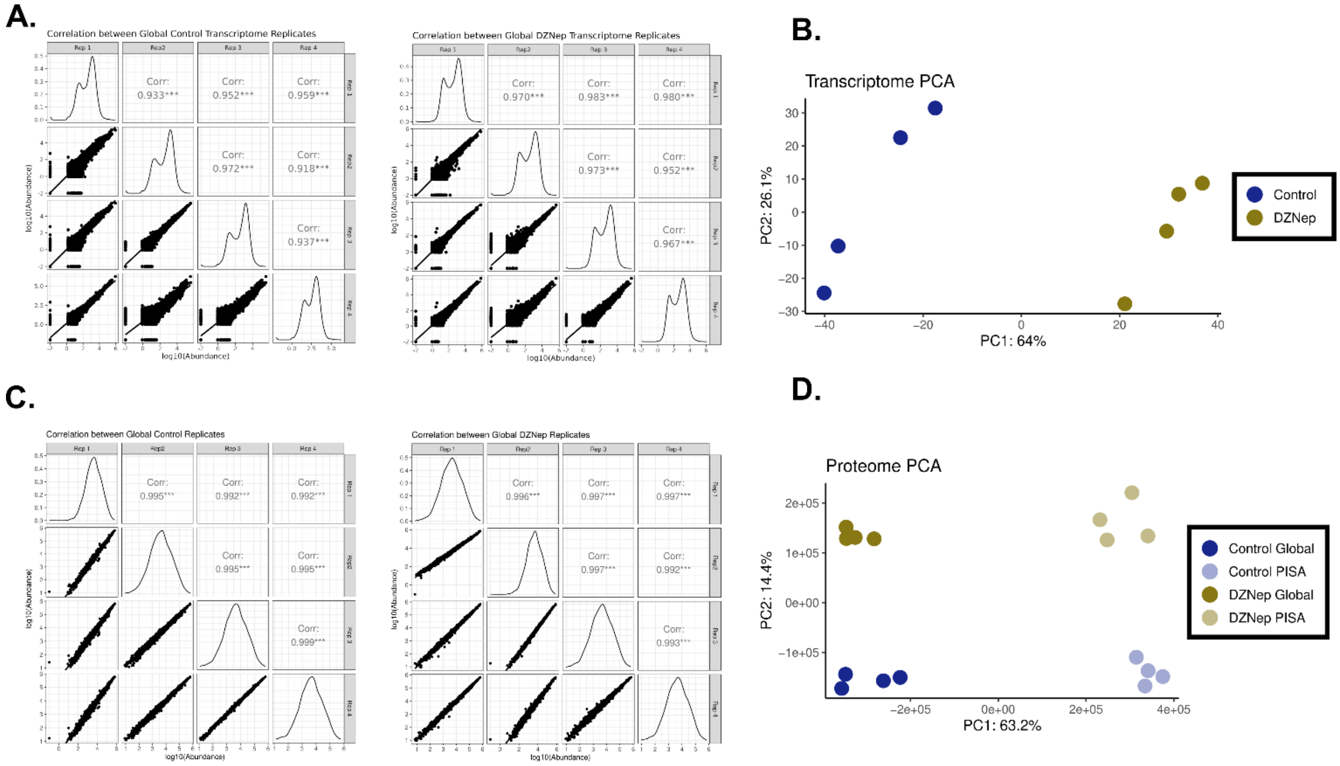

Figure S2: Transcriptome and proteome replicates show a high level of reproducibility.

Pearson correlations between the (a) RNAseq biological samples (n = 19,051) and (c) global proteome biological samples (n = 3,585)(Pearson correlation, ***p<0.001). Control biological replicates are on the left, and DZNep biological replicates are on the right. Principal component analysis of the (b) RNAseq samples and (d) global proteome and PISA samples. Dark blue dots represent the control global proteome biological replicates, and light blue dots represent the PISA biological replicates. Gold dots represent the DZNep-treated global proteome replicates, while the light-gold dots represent the PISA biological replicates.

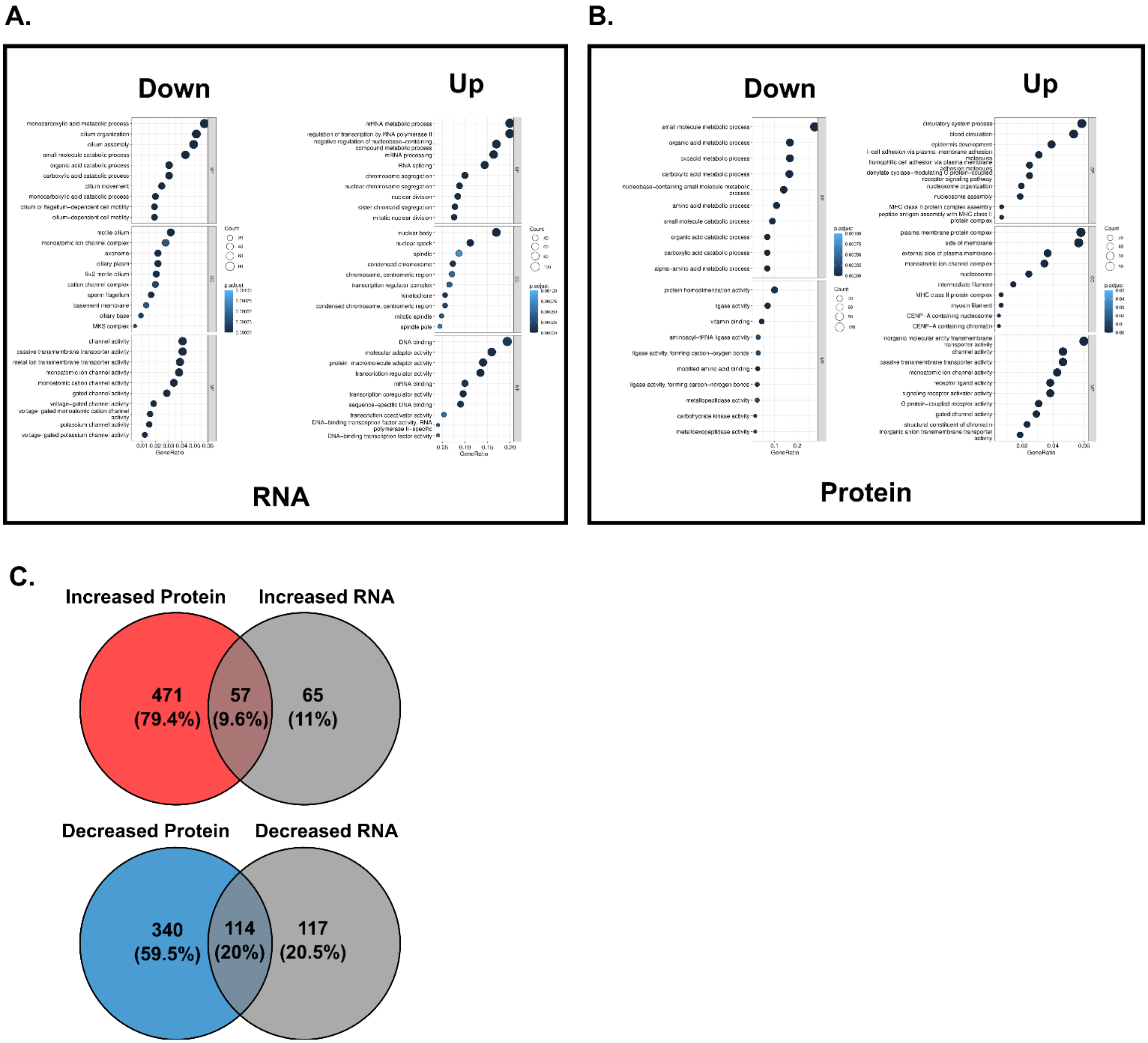

Figure S3: GO term analysis and correlation between RNAseq and proteome abundances.

Enriched GO terms of the (a) downregulated (left) and upregulated (right) transcripts or (b) decreased (left) and increased (right) proteins. Plots were made using clusterprofiler with the observed transcriptome as the background. Top 10 significant GO terms for each category (BP = biological function, CC = Cellular Component, and MF= molecular function; p-value < 0.05) are visualized. (c) Venn diagram of the (top) proteins with increased abundance and genes with upregulated transcripts and (bottom) proteins with decreased abundance and genes with downregulated transcripts.

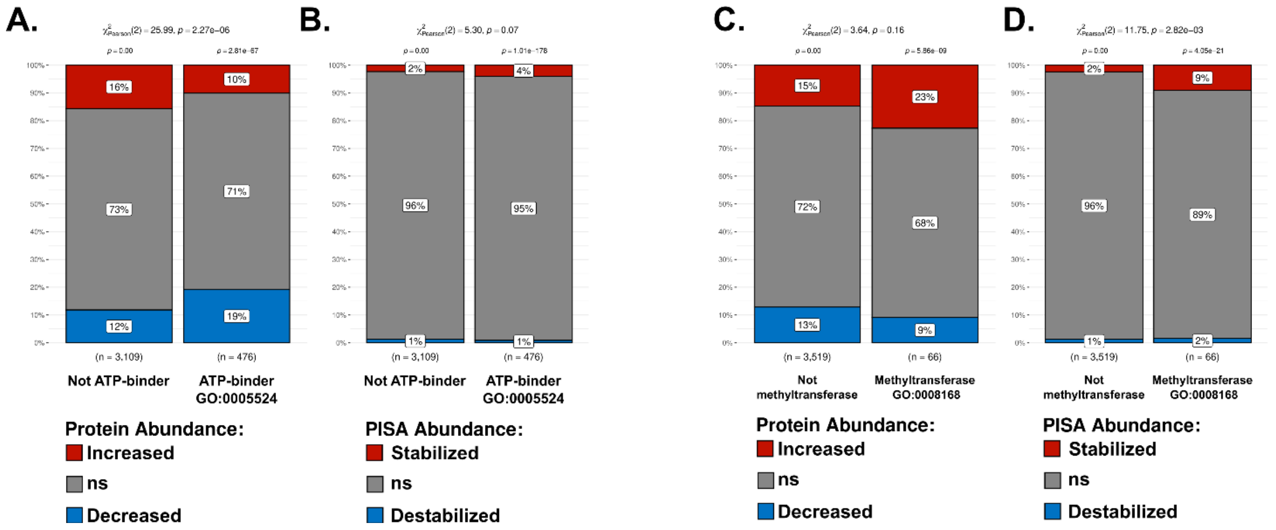

Figure S4: ATP binders and methyltransferases are more likely to have either altered protein abundance or thermal stability

(a & b) Proteins identified as ATP-binder using GO term GO0005524. A Chi-square analysis was conducted between the ATP-binder category (ATP-binder or not-ATP binder) and either (a) the protein abundance category (Increased, Decreased, or not significant) or (b) the PISA thermal stability category (Stabilized, Destabilized, or not significant).

(c & d) Proteins identified as a methyltransferase using GO term GO0008168. A Chi-square analysis was conducted between the methyltransferase category (methyltransferase or not methyltransferase and either (c)the protein abundance category (Increased, Decreased, or not significant) or (d) the PISA thermal stability category (Stabilized, Destabilized, or not significant).

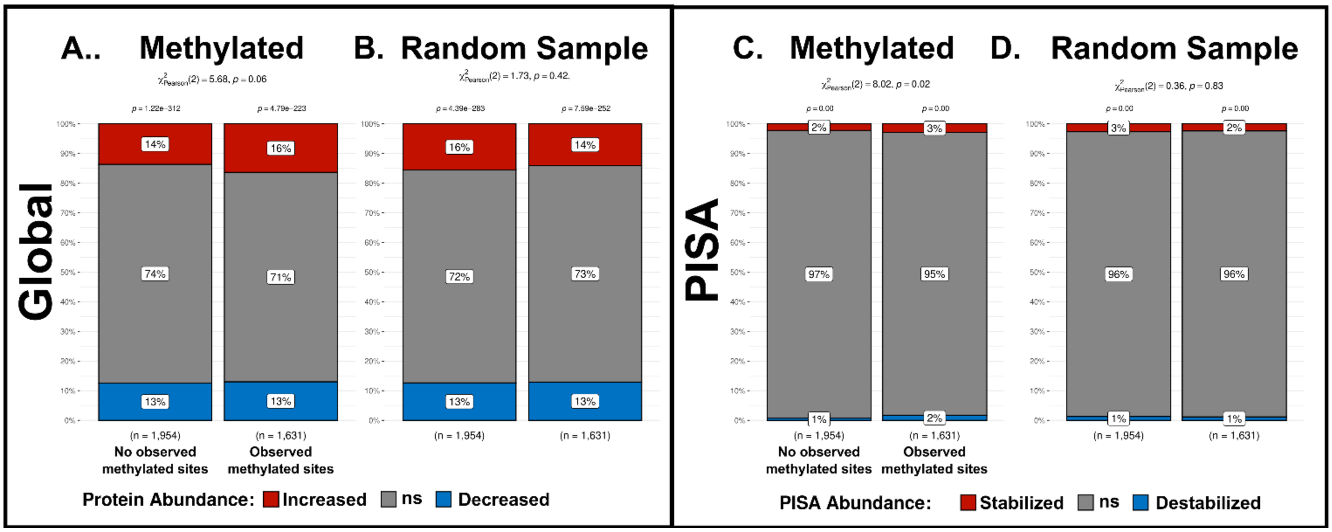

Figure S5: Methylated proteins are affected by DZNep treatment.

PhosphoSite Plus and Berryhill et al. 2023 were used to identify proteins with an observed lysine methylated site. A random sample containing the same number of proteins was used as the random control (n = 1954). Chi-square analysis was conducted between the methylated category (observed methyl site or no observed methyl site) and the (a & b) protein abundance category (Increased, Decreased, or not significant) or the (c & d) PISA thermal stability category (Stabilized, Destabilized, or not significant).
